# Understanding Collaborative CT and MRI Utilization Through Network Analysis: Retrospective Study Using Administrative Claims Data

**DOI:** 10.2196/72248

**Published:** 2026-04-23

**Authors:** Tomoki Ishikawa, Jumpei Sato, Yasuhiro Morii, Masaru Kitsuregawa, Kazuo Goda, Katsuhiko Ogasawara, Naohiro Mitsutake

**Affiliations:** 1 Institute for Health Economics and Policy Minato-ku, Tokyo Japan; 2 Department of Social Medicine Asahikawa Medical University Asahikawa, Hokkaido Japan; 3 Faculty of Health Sciences Hokkaido University Sapporo Japan; 4 Institute of Industrial Science The University of Tokyo Tokyo Japan; 5 Center for Outcomes Research and Economic Evaluation for Health National Institute of Public Health Wako Japan; 6 Faculty of Engineering Muroran Institute of Technology Muroran, Hokkaido Japan

**Keywords:** network analysis, administrative data, diagnostic imaging, computed tomography, magnetic resonance imaging, health care resource management, interinstitutional cooperation, shared use

## Abstract

**Background:**

Japan has one of the highest densities of computed tomography (CT) and magnetic resonance imaging (MRI) scanners globally, yet efficient resource allocation remains a challenge amid demographic shifts and regional health care disparities.

**Objective:**

This study aimed to develop an analytic framework using network analysis techniques to understand the collaborative use of CT and MRI devices across health care facilities in a Japanese prefecture.

**Methods:**

A retrospective observational study was conducted using outpatient receipt data from Japan’s National Health Insurance and the Late-Stage Elderly Medical System, covering fiscal years 2016 to 2019. Network analysis techniques were used to identify patterns of shared use among medical institutions. Network graphs with community detection were developed to visualize collaborative relationships, and density and reciprocity metrics were calculated to assess interinstitutional cooperation.

**Results:**

CT examinations increased from 287,782 (2016) to 307,029 (2019), while MRI examinations increased from 107,876 to 115,929 over the same period. Collaborative examinations also increased for both modalities. Network density remained relatively stable (CT: 3.10-3.50×10^-3^; MRI: 3.20-3.70×10^-3^), while reciprocity decreased (CT: 9.74×10^-2^ to 7.79×10^-2^; MRI: 2.82×10^-2^ to 1.56×10^-2^). Community detection analysis showed differences in the distribution of medical institutions across clusters over time.

**Conclusions:**

Network analysis revealed structural changes in collaborative CT and MRI use patterns, including declining reciprocity, which suggests a shift toward more unidirectional referral patterns. This analytic framework provides a method for health care planners to assess interinstitutional collaboration and inform resource allocation strategies for shared diagnostic equipment.

## Introduction

### Background

Japan has one of the highest densities of computed tomography (CT) and magnetic resonance imaging (MRI) scanners per capita in the world, significantly surpassing other resource-limited countries such as the United States and European nations. According to the Organisation for Economic Co-operation and Development (OECD) data, Japan’s health care system has more than 153 CT scanners and 57 MRI units per million population [1]. These figures are more than twice the OECD average. This high density is attributed to factors such as an aging population, strong demand for advanced diagnostic imaging, and policies that facilitate investment in medical technology. However, despite the abundance of these devices, disparities in access and underutilization in certain regions persist, highlighting the need for an efficient resource allocation strategy. In Japan, given the anticipated population decline [2], the government has been implementing policies to encourage the shared use of CT and MRI equipment among regional medical facilities and patient referrals [3,4].

In Japan, prefectural governments are mandated under the Medical Care Act to develop strategic plans for the shared use of diagnostic devices to optimize the allocation of expensive medical equipment [5]. The efficient sharing of such devices among medical facilities is crucial for enhancing health care accessibility and reducing health care costs. Formulating effective plans requires a comprehensive understanding of the current cooperation among health care facilities. Unfortunately, publicly available statistical data offer limited insights into the current actual shared-use patterns and interinstitutional collaborations related to diagnostic devices.

Formulating effective plans for shared equipment use requires empirical evidence on current collaborative patterns among health care facilities. Without systematic data on how facilities actually share diagnostic imaging resources, prefectural governments must rely on incomplete information when making equipment allocation decisions. This information gap can lead to imbalances in the distribution of resources, ultimately affecting patient access to timely diagnostic services. An analytic framework that can quantify and visualize interinstitutional collaboration patterns would therefore provide an evidence base for health care resource planning.

### Literature Review

Some recent studies have suggested the potential benefits of shared use of high-cost diagnostic equipment such as CT and MRI in health care systems. Some research indicates that increased use of CT and MRI correlates with hospital cost inefficiency [6]. The strategic sharing of these devices within regional health care networks can lead to significant cost savings and improvements in service delivery [7]. In medium to large hospitals, the introduction of CT and MRI contributes to improved quality and resource efficiency, but in smaller clinics, the use of diagnostic imaging may not contribute to these improvements [8]. Therefore, appropriate allocation of medical equipment, considering hospital volume and establishing a coordinated method of shared use, could improve cost efficiency.

To accurately depict real-world scenarios using empirical data and to formulate evidence-based policies, there remains a notable absence of studies addressing analytic methods for collaborative medical equipment use.

The aim of this study was to propose an analytic framework for understanding the collaborative use of CT and MRI devices across health care facilities of diverse scales. Specifically, we used network analysis techniques—including network graph visualization, density and reciprocity metrics, and community detection—to characterize the sharing dynamics of CT and MRI equipment within a regional health care network. Through analysis of longitudinal administrative claims data, we aimed to describe patterns of collaborative use that may inform resource allocation decisions.

## Methods

### Data Sources

This study used an outpatient receipt database from the National Health Insurance and Health Insurance System for people aged 75 years and older in a Japanese prefecture [9]. Receipt databases provide detailed records of health care services, including the history of imaging examinations, patient demographics, and medical institution identifiers, which could be used as a resource for analyzing interinstitutional collaboration. Our analysis covered the fiscal years 2016 to 2019 to capture longitudinal changes and identify potential factors influencing collaboration.

This study used a retrospective observational design to examine the trends and patterns in diagnostic device sharing. The procedure in this study involved five key processes: (1) extracting the study population from receipt data, (2) identifying the shared use of CT and MRI for these patients, (3) identifying the relationship between collaborative medical facilities engaged in shared use, (4) constructing network graphs of collaboration based on a network analysis method, and (5) evaluating the collaborative structure by calculating network measurements.

### Study Population

The targeted patients were covered by the National Health Insurance and the Late-Stage Elderly Healthcare System and underwent CT and MRI examinations between fiscal years 2016 and 2019. The inclusion criteria required patients to have undergone at least one CT or MRI scan, maintained continuous insurance coverage throughout the survey period, and had sufficient outpatient consultation records for tracking health care use patterns. Patients who discontinued insurance during the period of shared equipment use or failed to follow medical advice regarding facility visits were excluded from the study population.

### Analyzing Methods

Network analysis is beginning to be recognized as an important approach for understanding and improving large-scale health system operations, with its full potential yet to be established [10,11]. Previous research using the same regional claims database demonstrated the use of network analysis for evaluating patient-sharing structures among medical facilities [12]. Traditional analytic approaches, such as descriptive statistics and regression analyses, typically treat medical facilities as independent units, limiting their ability to capture interinstitutional relationships [13,14]. Network analysis addresses this limitation by enabling the examination of relational structures among health care providers [15]. Network metrics, such as density and reciprocity, quantify connectivity patterns, while community detection algorithms can identify clusters of collaborating facilities [16,17].

This approach is used to evaluate the types and structures of interactions between medical facilities and network indicators to identify the real-world correlations of these network indicators [18]. In this study, the networks were analyzed using adjacency matrices, where nodes represent medical facilities and directed edges indicate the relationships for the shared use of diagnostic equipment.

In this study, we defined “shared use” medical cooperation as follows:

- Condition 1: a medical facility conducts only a single diagnostic imaging visit within a continuous 3-month period
- Condition 2: another medical facility conducts multiple visits within a continuous 6-month period
- Condition 3: the date of the diagnostic imaging examination at the medical facility in condition 1 falls within the period of visits at the medical facility specified in condition 2.

This study identified medical facilities meeting the specified conditions solely for conducting examinations as a “target facility,” while those not conducting examinations but identified as requesting them were categorized as a “source facility.”

We organized consecutive medical consultations meeting these criteria chronologically, identified the originating and receiving medical facilities, and constructed an adjacency matrix. Subsequently, we performed network analysis using this matrix, where medical facilities were nodes and shared-use relationships were represented as directed edges. We depicted network graphs to visualize relationships between medical facilities with the Fruchterman-Reingold algorithm, which is commonly used to enhance the understanding of structural patterns [19]. We performed community detection using the Louvain algorithm, which optimizes modularity to identify densely connected groups of facilities within the broader network [18]. This algorithm iteratively assigns nodes to communities in a manner that maximizes the density of connections within communities relative to connections between communities. The resulting cluster groups represent naturally occurring collaborative communities—groups of medical facilities that share patients more frequently with each other than with facilities outside the cluster.

### Measurements

The key evaluation metrics used in this study were network density, reciprocity, and collaboration count (the number of shared use cases between facilities).

Network density and reciprocity were calculated using the following formulas:

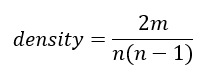

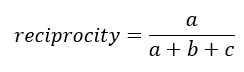





Here, *n* represents the total number of medical facilities (nodes) in the network, and m denotes the total number of shared-use relationships (edges) between facilities. Density represents the proportion of actual referral connections relative to all possible connections among facilities [11,20].

For the reciprocity formula, *a* denotes the number of bidirectional edges, where both facility A refers patients to facility B and facility B refers patients to facility A for shared imaging examinations within the same fiscal year. The terms *b* and *c* represent unidirectional edges: *b* denotes edges where only facility A refers to facility B and *c* denotes edges where only facility B refers to facility A [21]. Higher reciprocity indicates that facilities mutually refer patients to each other, while lower reciprocity indicates unidirectional referral patterns where patients are sent to specific facilities without reciprocal referrals.

These metrics are relative values; in this study, we focused on temporal changes in these indicators, using reciprocity to supplement the interpretation of changes in network density. Differences in facility characteristics (fiscal years 2016-2019) were compared using the Kruskal-Wallis test, while CT and MRI modalities were compared via the Mann-Whitney *U* test (2-sided *P*<.05). The analysis was conducted using R software (version 4.3.2; R Foundation for Statistical Computing) with specialized libraries for network analysis (igraph, networkD3, and ggraph).

### Ethical Considerations

Our study was approved by the Ethics Committee of the Institute for Health Economics and Policy (approval R4-010). The requirement for informed consent was waived because of the anonymized nature of the data in the database. Patient confidentiality was maintained throughout, and no compensation was provided to participants, as this was a retrospective analysis of existing administrative claims data.

## Results

**Figure 1:**
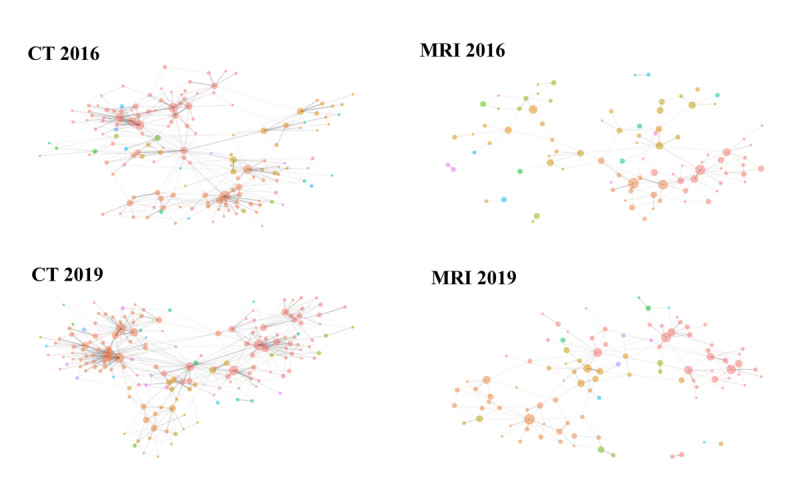
Computed tomography (CT) and magnetic resonance imaging (MRI) shared-use network graph (2016 to 2019).

Figure 1 depicts a network diagram illustrating the coordination of CT and MRI use. Table 1 summarizes annual statistics for CT examinations, coordinated CT examinations, and the proportion of coordinated CT use from fiscal years 2016 to 2019, with data provided for MRI in a similar manner.

**Table 1:** Trends in computed tomography (CT) and magnetic resonance imaging (MRI) examinations and collaborative use.

Year	CT examinations, n	Shared CT examination, n	Proportion of collaborative shared use	MRI examinations, n	Shared MRI examinations, n	Proportion of shared MRI use
2016	287,782	1507	0.524	107,876	537	0.498
2017	298,755	1665	0.557	112,018	560	0.500
2018	304,322	1580	0.519	114,528	574	0.501
2019	307,029	1774	0.578	115,929	612	0.528

From 2016 to 2019, CT examinations increased from 287,782 in 2016 to 307,029 in 2019. Collaborative CT examinations also increased from 1507 to 1774 over the same period, with the proportion of collaborative CT use ranging between 0.52 and 0.58. Similarly, MRI examinations increased from 107,876 in 2016 to 115,929 in 2019. Collaborative MRI examinations also increased from 537 to 612, with the proportion of collaborative MRI use ranging between 0.50 and 0.53.

The edges observed in Figure 1 compared with CT correspond to the count of source and target medical facilities in Table 2. CT requests and MRI requests are aggregated separately, focusing on both the number of requests made and received per medical facility. *P* values were calculated using the Kruskal-Wallis test for comparisons of facility characteristics across fiscal years 2016 to 2019.

**Table 2:** Trends in requests and requests received for shared use by source and target medical facilities from 2016 to 2019.

	2016	2017	2018	2019	*P* value
Source medical facilities (CT^a^)	531	529	530	530	—^b^
Number of CT requests, mean (SD)	2.47 (3.06)	2.70 (3.43)	2.63 (3.54)	2.73 (3.72)	.61
Number of beds, mean (SD)	32.87 (100.48)	31.89 (98.16)	36.06 (105.02)	32.88 (101.62)	.92
Target medical facilities (CT)	134	129	119	122	—
Number of CT requests received, mean (SD)	9.79 (18.81)	10.88 (18.45)	11.02 (19.37)	11.39 (20.99)	.92
Number of beds, mean (SD)	126.24 (168.52)	128.84 (169.54)	131.20 (169.85)	131.52 (173.42)	.99
Source medical facilities (MRI^c^)	281	279	281	283	—
Number of MRI requests, mean (SD)	1.61 (1.66)	1.71 (1.89)	1.75 (1.86)	1.74 (1.81)	.81
Number of beds, mean (SD)	30.86 (103.43)	39.22 (117.50)	38.60 (116.32)	37.88 (112.25)	.81
Target medical facilities (MRI)	75	72	65	66	—
Number of MRI requests received, mean (SD)	6.97 (6.98)	6.31 (7.23)	6.12 (7.27)	6.30 (7.47)	.92
Number of beds, mean (SD)	171.63 (179.47)	175.80 (172.87)	167.46 (174.59)	174.15 (175.92)	.99

^a^CT: computed tomography.

^b^Not applicable.

^c^MRI: magnetic resonance imaging.

Across the years from 2016 to 2019, facility-level characteristics showed no statistically significant year-to-year variation for CT (requests: *P*=.61; beds: *P*=.92) or MRI (requests: *P*=.81; beds: *P*=.81). The mean number of requests received per medical facility was higher for CT than for MRI. No statistically significant differences were observed in the mean number of hospital beds across years for either modality. Network density values for MRI were lower, approximately 2.50×10⁻^3^, 2.45×10⁻^3^, and 2.40×10⁻^3^, indicating a less connected network. Similarly, reciprocity declined slightly, with values around 7.50×10⁻^2^, 7.45×10⁻^2^, and 7.20×10⁻^2^ over the 3-year period, reflecting a weaker bidirectional collaboration compared with CT.

Table 3 shows the metrics for CT and MRI shared-use networks presented annually from 2016 to 2019. For CT networks, density showed slight variations year to year, ranging from 3.20×10^−3^ in 2017 to 3.50×10^−3^ in 2018. Reciprocity decreased over the years from 9.74×10^−2^ in 2016 to 7.79×10^−2^ in 2019. MRI networks exhibited different patterns. The density remained relatively stable, with values ranging from 3.40×10^−3^ to 3.20×10^−3^ across the years. Reciprocity declined from 2.82×10^−2^ in 2016 to 1.56×10^−2^ in 2019.

**Table 3:** The metrics for computed tomography (CT) and magnetic resonance imaging (MRI) shared-use networks from 2016 to 2019.

Year	CT network metrics			MRI network metrics	
	Density	Reciprocity	Density		Reciprocity
2016	3.30×10^−3^	9.74×10^−2^	3.40×10−3		2.82×10^−2^
2017	3.20×10^−3^	9.80×10^−2^	3.60×10^−3^		2.15×10^−2^
2018	3.50×10^−3^	8.78×10^−2^	3.70×10^−3^		1.77×10^−2^
2019	3.10×10^−3^	7.79×10^−2^	3.20×10^−3^		1.56×10^−2^

**Figure 2:**
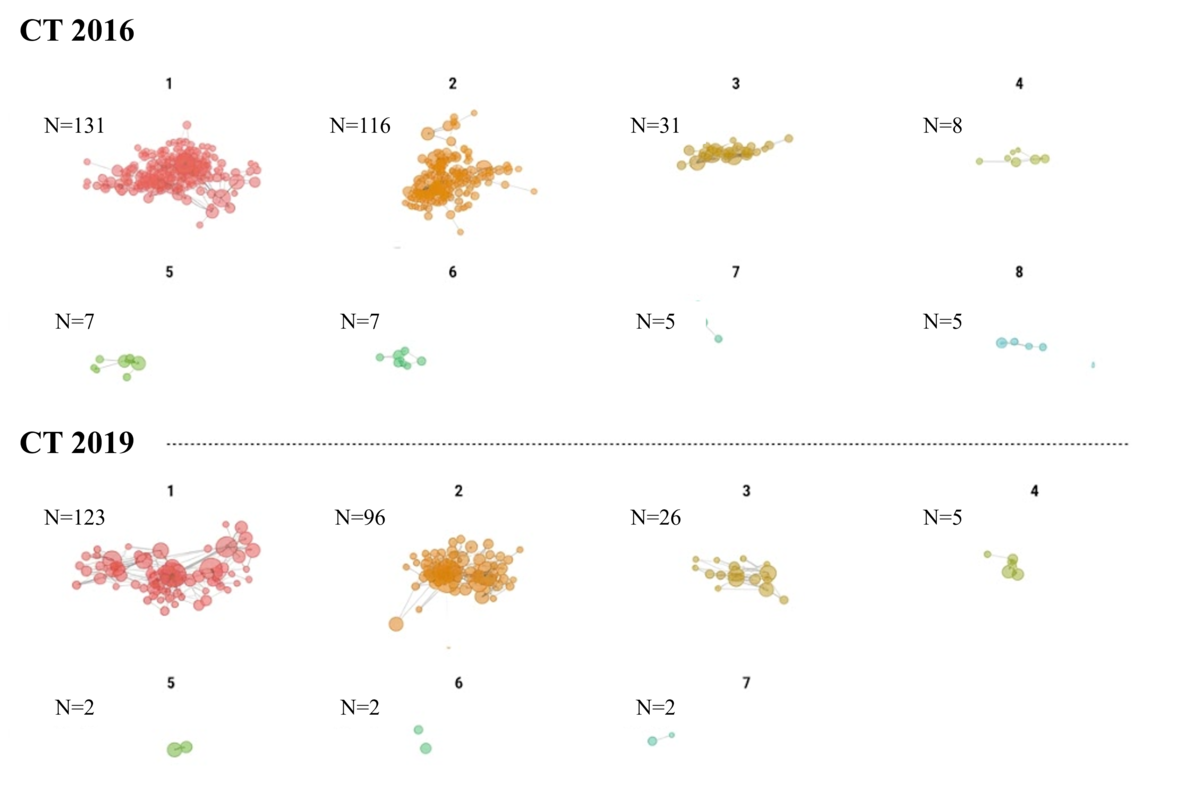
Clustered network graph of computed tomography (CT) shared use from 2016 to 2019 (with numerical IDs for each cluster group).

**Figure 3:**
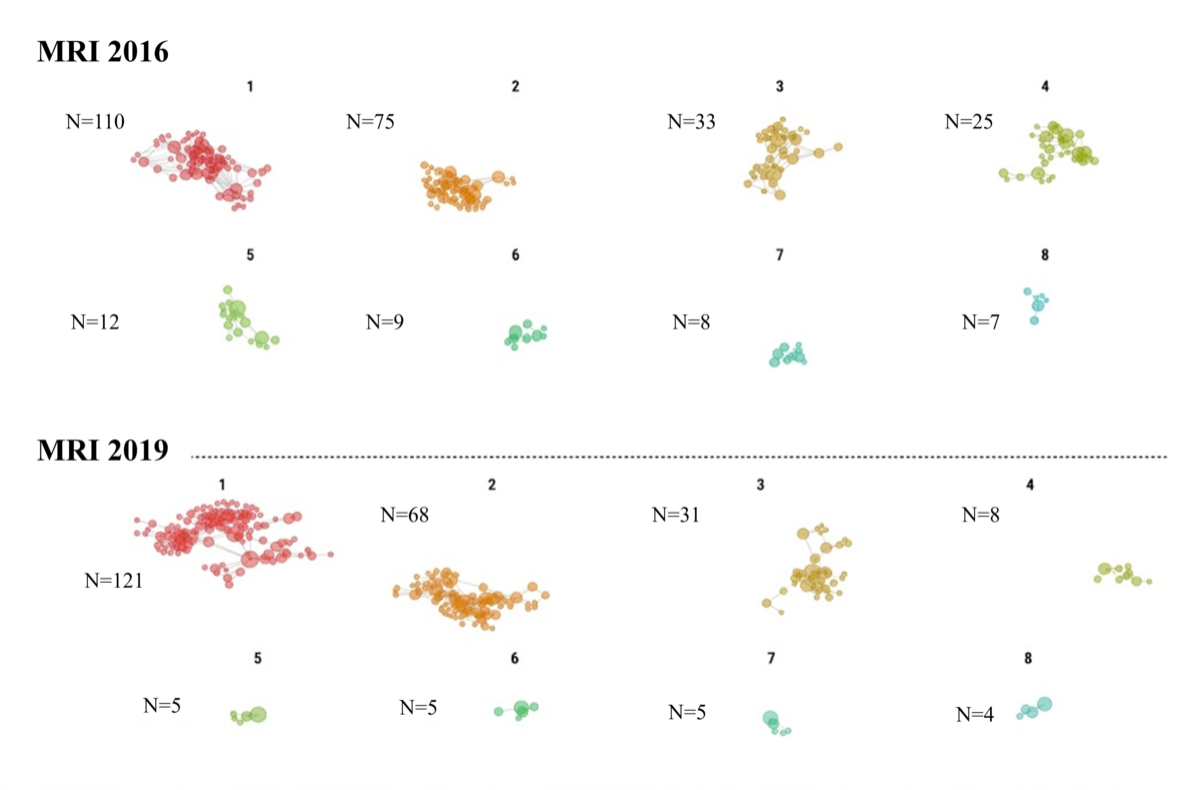
Clustered network graph of magnetic resonance imaging (MRI) shared use from 2016 to 2019 (with numerical IDs for each cluster group).

Figures 2 and 3 illustrate the network of medical institutions categorized by clusters based on the Louvain community detection algorithm. These communities were visually distinguished using different colors in the network diagrams. The cluster numbers (1-8) are arbitrary identifiers assigned by the algorithm; however, the spatial arrangement in the figures reflects the relative strength of connections between facilities, with more closely positioned facilities demonstrating stronger collaborative relationships. The distribution of medical institutions across clusters has shown changes from 2016 to 2019, indicating a concentration of institutions within specific clusters.

## Discussion

### Principal Findings

This study investigated the collaborative use patterns of CT and MRI within a regional health care network from 2016 to 2019. The annual trends in examination volumes and collaborative use proportions were analyzed to provide insights into the operational dynamics of these diagnostic modalities.

CT examinations increased from 287,782 in 2016 to 307,029 in 2019. Collaborative CT examinations also increased from 1507 to 1774, with the proportion of collaborative CT use ranging between 0.524 and 0.578 over the study period. Similarly, MRI examinations increased from 107,876 in 2016 to 115,929 in 2019. Collaborative MRI examinations increased from 537 to 612, with the proportion of collaborative MRI use ranging from 0.498 to 0.528. The network structural characteristics of this shared use are examined in the following paragraphs.

The analytic framework used in this study demonstrates the value of network analysis for health care resource management research. Prior studies examining medical equipment use have primarily relied on facility-level statistical analyses, which cannot reveal the structural patterns of interinstitutional collaboration [13,14]. Our network-based approach addresses this limitation by enabling systematic assessment of how shared-use relationships form, evolve, and concentrate over time [15,16].

Simple counts of collaborative examinations (Table 1) indicate increasing collaborative activity; however, network metrics (Table 3) reveal that this growth occurs within an increasingly concentrated network structure characterized by declining reciprocity. This pattern would not be apparent from traditional analytic approaches that do not account for the relational nature of health care collaboration.

Our findings illustrate how network analysis can provide information relevant to resource allocation planning. The decreasing reciprocity observed for both CT and MRI networks, combined with changes in cluster composition identified through community detection, describes an evolving pattern of collaborative use that would not be captured by conventional facility-level statistics. For health care planners developing prefectural strategic plans for shared equipment use, these network metrics can serve as supplementary information sources: density measurements can describe the extent of collaborative relationships within a region, reciprocity can indicate the directionality of referral patterns, and community detection can identify naturally occurring collaborative groupings that may inform the geographic planning of equipment investment.

Our findings provide several insights into the collaborative CT and MRI use patterns through a network-based analytic framework, offering perspectives that extend beyond traditional use statistics. Examination volumes increased for both modalities over the study period, possibly reflecting changing demand and access to diagnostic imaging services. The proportion of collaborative CT and MRI examinations also increased, suggesting evolving coordination and resource sharing among medical facilities. This aspect underscores the strength of our study in quantifying collaborative practices, which are crucial for optimizing resource allocation and enhancing diagnostic efficiency.

There were no significant changes in the number of source medical facilities for either CT or MRI use. Conversely, target medical facilities exhibited a decrease. This suggests a potential concentration of diagnostic imaging functions within facilities that perform these examinations. The clustering of medical facilities into specific clusters as depicted in Figure 2 supports this pattern of unidirectional referral within the network.

The stable facility-level characteristics (Table 2; all *P*>.05) suggest that collaborative use practices remained within a stable institutional environment over the study period. However, it is important to note that the rate of shared use remains relatively small compared to the total number of tests conducted. This indicates the potential for expansion and further integration of shared imaging services in the future. The higher mean number of CT requests received per facility, compared with MRI, may reflect the broader clinical indications and versatility of CT imaging in routine clinical practice.

The network metrics reveal distinctive patterns between CT and MRI networks, particularly in terms of density and reciprocity, indicating differing levels of interconnectivity and collaborative interaction within the health care network. Reciprocity, which measures bidirectional referrals between medical facilities, decreased in both CT and MRI networks from 2016 to 2019. This decrease suggests a shift toward more unidirectional referral patterns, possibly influenced by evolving clinical practices or logistical challenges specific to each imaging modality. The lower reciprocity in MRI networks, despite relatively stable densities, might indicate a less symmetrical exchange of referrals compared with CT. This asymmetry could stem from specialized clinical needs or operational factors unique to MRI, such as availability, cost considerations, or specific clinical indications.

While our empirical findings are specific to the Japanese health care context, the analytic framework itself has broader international applicability. The network analysis methods we used—including density calculation, reciprocity measurement, and community detection—are methodologically universal and can be applied to any health care system with appropriate administrative data on patient referrals or facility use. Several contextual factors specific to Japan should be considered when interpreting our findings or adapting this framework to other settings. Japan maintains an exceptionally high density of CT and MRI equipment, approximately 4 times the OECD average [1]. This high density creates an environment where shared-use networks can form extensively, as numerous facilities possess equipment that could function as either referral sources or referral destinations. Health care systems with lower equipment density would likely demonstrate different network structures, potentially showing higher concentration from the outset. Japan’s universal health insurance system and comprehensive claims database (National Database of Health Insurance Claims) provide the detailed use records that enable this type of analysis. Countries with similar insurance-based systems and administrative databases—such as Germany, South Korea, Taiwan, and France—could readily adapt this methodology. However, health care systems with fragmented insurance coverage or limited administrative data infrastructure may face challenges in constructing comparable datasets. Additionally, Japan’s relatively unrestricted patient access to health care facilities (free access system) influences referral patterns differently than countries with formal gatekeeping systems. In systems requiring primary care referrals for specialist services, network structures would inherently show more hierarchical patterns. Despite these contextual variations, the core analytic approach—using network metrics to quantify interinstitutional collaboration and identify structural changes over time—provides a transferable framework for health care system analysis across diverse national contexts.

While our study provides valuable insights, it is important to acknowledge several limitations. First, our results are specific to a single prefecture in Japan where CT and MRI facilities are densely concentrated. This geographic specificity may restrict the generalizability of our findings to regions with different health care infrastructures and resource distributions.

Second, the data used in this study are derived from insurance reimbursement records, reflecting actual clinical use but potentially excluding examinations conducted outside insurance claims (eg, health screenings and private pay services). This limitation implies that our findings may not fully capture the comprehensive use of CT and MRI services in the region.

Third, the identification of shared use is based on specific criteria applied in this study. Variations in these criteria could potentially impact the results, influencing the interpretation of collaborative patterns and the efficiency of resource allocation across medical institutions. Fourth, our network analysis captures structural relationships but cannot directly assess causality or the quality of care delivered through these collaborative arrangements. In summary, these limitations are acknowledged; however, our study’s approach supports the development of methodological approaches without compromising the interpretation of collaborative health care practices and resource management.

### Conclusions

Using the receipt database from 2016 to 2019, our analysis showed increases in both CT and MRI examinations, accompanied by increases in shared use. Network analysis revealed decreasing reciprocity in both CT and MRI networks, suggesting a shift toward more unidirectional referral patterns. Moreover, we observed a concentration of shared-use requests among specific medical institutions within the shared-use network structure. These findings enabled us to propose an analytic framework grounded in network analysis theory for assessing the practical dynamics of resource sharing, including medical equipment, across health care settings. This framework may inform prefectural strategic plans for shared equipment use as mandated under the Medical Care Act.

## Data Availability

The data are not publicly available due to restrictions imposed by the regional insurers and local governments. The dataset was provided under contractual agreements prohibiting third-party sharing. Requests for access may be directed to the corresponding author.

## Abbreviations

CT: computed tomography
MRI: magnetic resonance imaging
OECD: Organisation for Economic Co-operation and Development
